# *In Silico* Screening, Genotyping, Molecular Dynamics Simulation and Activity Studies of SNPs in Pyruvate Kinase M2

**DOI:** 10.1371/journal.pone.0120469

**Published:** 2015-03-13

**Authors:** Ponnusamy Kalaiarasan, Bhupender Kumar, Rupali Chopra, Vibhor Gupta, Naidu Subbarao, Rameshwar N. K. Bamezai

**Affiliations:** 1 School of Biotechnology, Shri Mata Vaishno Devi University, Katra, Jammu and Kashmir, India; 2 National Centre of Applied Human Genetics, School of Life Sciences, Jawaharlal Nehru University, New Delhi, India; 3 School of Computational and Integrative Sciences, Jawaharlal Nehru University, New Delhi, India; Aligarh Muslim University, INDIA

## Abstract

Role of, 29-non-synonymous, 15-intronic, 3-close to UTR, single nucleotide polymorphisms (SNPs) and 2 mutations of Human Pyruvate Kinase (PK) M2 were investigated by *in-silico* and *in-vitro* functional studies. Prediction of deleterious substitutions based on sequence homology and structure based servers, SIFT, PANTHER, SNPs&GO, PhD-SNP, SNAP and PolyPhen, depicted that 19% emerged common between all the mentioned programs. SNPeffect and HOPE showed three substitutions (C31F, Q310P and S437Y) *in-silico* as deleterious and functionally important. *In-vitro* activity assays showed C31F and S437Y variants of PKM2 with reduced activity, while Q310P variant was catalytically inactive. The allosteric activation due to binding of fructose 1-6 bisphosphate (FBP) was compromised in case of S437Y nsSNP variant protein. This was corroborated through molecular dynamics (MD) simulation study, which was also carried out in other two variant proteins. The 5 intronic SNPs of PKM2, associated with sporadic breast cancer in a case-control study, when subjected to different computational analyses, indicated that 3 SNPs (rs2856929, rs8192381 and rs8192431) could generate an alternative transcript by influencing splicing factor binding to PKM2. We propose that these, potentially functional and important variations, both within exons and introns, could have a bearing on cancer metabolism, since PKM2 has been implicated in cancer in the recent past.

## Introduction

Pyruvate kinase (PK) is a rate-limiting glycolytic enzyme which irreversibly transfers phosphate group from phosphorenolpyruvate (PEP) to adenosine diphosphate (ADP) producing pyruvate and adenosine triphosphate (ATP). In mammals, PK is expressed in four different isoforms, L, R, M1 and M2, depending on the types of tissues [1]. Pyruvate Kinsae (PK) M2 is exclusively expressed in the fetus, adult dividing and tumor cells [2]. PKM gene encodes two isoforms, M1 and M2, following alternative splicing. The two isoforms differ by 22 amino acids. M1 is a non-allosteric isoform, and the PKM2 an allosterically regulated by Fructose 1, 6-bisphosphate (FBP) [3]. It is a homo-tetrameric protein where each monomer consists of four domains A, B, C and N. The structural features of these domains demonstrate that A-domain contains amino acid residues between 44–116 and 219–389 with an *α8/β8* barrel tertiary structure motif. B-domain consists of residues Pro 117 to Pro 218 with a combination of β-sheets and random coils. Within the cleft formed between the A and B domains lies the active site (PEP binding site); and the FBP-binding site is positioned within the C-domain, constituted of residues 390–531. The N-terminus with residues 1–43, forms a small domain, consisting of helix-turn-helix motif [4]. The stretch of amino acids, which differentiates PKM2 from PKM1 protein, is located in the inter-subunit-contact-domain (ISCD), where two rare mutations (H391Y and K422R) in PKM2 have been reported in the Bloom syndrome background [5]. These mutations were observed to impact enzymatic activity, affinity for the substrate and the protein dynamicity, resulting in complete loss of allostericity in H391Y mutant [6]. Functionally, these mutations promoted cellular growth and polyploidy in *in vitro* experiments [7], suggesting their proposed role in cancer promotion [8,9], especially in Bloom syndrome patients besides affecting genomic stability [9].

The study of PKM2 is important because of its novel role unraveled in recent past in human cancers [10,11]. Increased expression of PKM2 has been reported in blood, prostate, breast, lung, colon, cervix, gastric and other cancers [12–15]. An increased utilization of glucose via glycolysis is a common phenotype of cancerous cells which is defined as the Warburg effect [16]. Pyruvate kinase M2 promotes the Warburg effect and tumor cell growth [12] and has been reported to interact with Hypoxia-inducible factor (HIF)-1alpha in the nucleus to act as a transcriptional co-activator to stimulate the expression of HIF-1 target genes, including Solute carrier family 2, facilitated glucose transporter member 9 (SLC2A). The potential of PKM2 and not PKM1 to mediate the Warburg effect was suggested when the alternatively spliced isoform (PKM1) from the PKM gene, failed to activate HIF-1 in cancer cells [17]. Nuclear PKM2 has also been reported to phosphorylate signal transducer and activator of transcription 3 (STAT3), which stimulates Mitogen-activated protein kinase kinase 5 (MAP2K5/MEK5) gene and enhances cell proliferation [18]. Despite a large amount of information generated for the critical role of PKM2 in sustaining cancers, it is surprising to find scanty information available on the role of the natural germline variations within this glycolytic pathway enzyme, which could affect its function with obvious implications in understanding cancer metabolism.

It was pertinent, therefore, to understand the functional impact of amino acid replacement, either due to mutation or non-synonymous variation on PKM2 protein expression and structure, bioinformatically and experimentally. Also, to unravel the possible role of intronic variations possibly resulting in splice variants, and affecting cellular metabolism through aberrant PKM2 protein function.

## Materials and Methods

**Fig 1:**
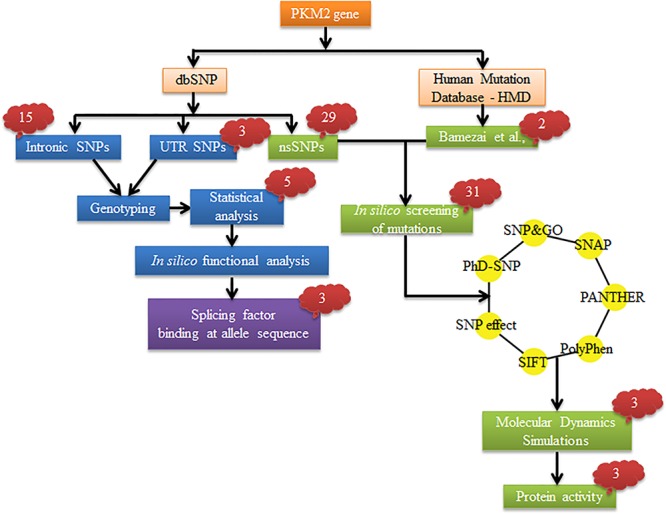
Schematic representation of the study. SNPs was categorized based on their location in the gene. The nsSNPs and mutation were analyzed together.

The data mining for SNPs in Human PKM2 was carried out on dbSNP135 databases (https://www.ncbi.nlm.nih.gov/SNP/). Mutation information of the gene was retrieved from Human mutation database. A total of 49 variations was selected in PKM2, which included 29 nsSNPs, 15 intronic, 3 close to UTR (2–5’UTR and 1–3’UTR), and 2 mutations, the latter reported by us for the first time [5]. Fig. 1 provides the detailed work flow of the study; where SNPs were selected by prioritization based on their minor allele frequency (>5%) in the publicly available dataset from the National Centre for Biotechnology Information (NCBI) Enterz SNP and the International HapMap project:Han Chinese, Japanese (Asian populations), and African (Ancesteral) populations. Few of the SNPs were included on their presence in the promoter, exonic, intronic-boundary, or unstranslated regions (UTRs), covering 2 kb upstream and downstream of the PKM2 gene. The sequence for all SNPs was downloaded from the dbSNP database in NCBI before the assay design.

### Prediction of deleterious and disease causing nsSNPs

We used Sorting Intolerant From Tolerant (SIFT), version 2 (http://blocks.fhcrc.org/sift/SIFT.html), a program which predicts the tolerant and deleterious substitutions within a given sequence [19]. Here, we provided PKM2 protein sequence with 31 substitutions. SIFT searched for similar sequences using database SWISS-PROT 51.3 and TREMBL 34.3, by selecting median conservation sequence score of 3.00. PolyPhen (Phenotyping Polymorphism) software version 2.2.2 (http://genetics.bwh.harvard.edu/pph2/) [20] was used to predict the consequence of an amino acid change on the structure and function of a protein on specific empirical rules. PolyPhen server exploits both UniProtKB/ UniRef100 non-redundant protein sequence and PDB/DSSP protein structure databases. PolyPhen input option requires a protein sequence, accession number P14618 combined with sequence position with amino acid variants AA1 (wild) and AA2 (mutant). We classified PolyPhen results into six categories based on the output of HumDiv and HumVar. The six categories were as follows: (i) highly damaging: both datasets (HumDiv and HumVar) predicted as probably damaging, (ii) damaging: anyone dataset predicted as probably damaging or possibly damaging, (iii) slightly damaging: both datasets predicted as possibly damaging, (iv) slightly tolerant: anyone dataset predicted as probably damaging or begin, (v) tolerant: anyone dataset predicted as possibly damaging or begin, (vi) highly tolerant: both datasets predicted as begin. PolyPhen was used for sequence and tetramer structure of PKM2 for prediction score of nsSNPs and mutations. PANTHER (Protein Analysis Through Evolutionary Relationships) (http://www.pantherdb.org/tools/csnpScoreForm.jsp) was used to estimate the likelihood of a particular non-synonymous (amino-acid changing) coding SNP to cause a functional impact on the protein. It calculates the substitution position-specific evolutionary conservation (subPSEC) score based on an alignment of evolutionarily related proteins [21]. The probability that a given variant would cause a deleterious effect on protein function was estimated by P_deleterious_, such that a subPSEC score of-3 corresponds to a P_deleterious_ of 0.5 [22]. The subPSEC score is the negative logarithm of the probability ratio of the wild-type and mutant amino acid at a particular position. PANTHER subPSEC scores are continuous values from 0 (neutral) to about-10 (most likely to be deleterious). PhD-SNP (http://snps.biofold.org/phd-snp/phd-snp.html), SNP&GO (http://snps-and-go.biocomp.unibo.it/snps-and-go/) are the methods based on SVMs that predicted disease related mutations from a protein sequence and structure respectively. SNP&GO required protein sequence to predict human disease-related mutations [23]. In PhD-SNP we used sequence and profile-based prediction [24]. SNAP (https://rostlab.org/services/snap/) predicted the functionality of mutated proteins based on neural-network method [25]; and required protein sequence and mutation residue with its position as an input. It used protein information, like secondary structure, conservation, solvent accessibility to predict the role of each mutation. SNPeffect 4.0 (http://snpeffect.switchlab.org/) [26] was used for phenotyping human SNPs. It provides four variant analyses, which include TANGO, WALTZ, LIMBO and FoldX. TANGO algorithm searches for cross-β aggregation within peptide sequences as well as in denatured proteins. WALTZ is an algorithm that accurately and specifically predicts amyloid-forming regions in protein sequences. It is more specific in terms of aggregate morphology than TANGO. LIMBO is a chaperone binding site predictor for the Hsp70 chaperones. The FoldX uses an atomic description of protein structure and provides a quantitative estimation of important interactions contributing to the protein stability. In the prediction of PKM2 nsSNPs, SNPeffect considered tetramer form of PKM2. The tetramer structure of non-synonymous single variant proteins (nsSVPs) along with wild-type PKM2 was generated using WHATIF server (http://swift.cmbi.ru.nl/servers/html/index.html) [27] and energy minimized using GROMACS. The stability of both tetramer structures was calculated using FOLDX (http://foldx.crg.es/) [28].

### Molecular dynamics simulations

The calculations were performed with GROMACS 4.5.3 package [29,30], using the GROMOS 96 force field. The box dimensions ensured that any protein atom was at least 1.5 nm away from the wall of the box with periodic boundary conditions and solvated by simple point charge (spc) [31] water molecules. NaCl counter ions were added to satisfy the electro-neutrality condition. Energy minimization was carried out using the steepest descent method. Berendsen temperature coupling [32] and Parrinello-Rahman pressure coupling [33] were used to keep the system in a stable environment (300 k, 1 bar), and the coupling constants were set to 0.1 and 2.0 ps for temperature and pressure, respectively. The partial mesh Ewald (PME) algorithm [34] was employed for electrostatic and Van der Waals interactions; cut-off distance for the short-range VdW (rvdw) was set to 1.4 nm, where Coulomb cut-off (r coulomb) and neighbour list (rlist) were fixed at 0.9 nm. All the bond lengths were constrained using the LINCS algorithm [35], and the time step was set to 0.002 ps. The complexes in a medium were equilibrated for 100 ps in NPT and NVT ensembles, respectively. Finally, a 95 ns molecular dynamics simulation was carried out for both wild and nsSVP complexes. All trajectories were stored every 2 ps for further analysis.

### Analysis of Molecular Dynamics Simulations

Structural properties of the wild and nsSVPs (C31F, Q310P and S437Y) of PKM2 were calculated from the trajectory files with the built-in functions of GROMACS 4.5.3. Structural analysis, such as root mean-square deviation (RMSD) and root-mean square fluctuation (RMSF) and radius of gyration were analyzed through the use of g_rmsd, g_rmsf and g_gyrate respectively, with the built-in functions of GROMACS. The number of hydrogen bond formed within the protein during the simulation was calculated using g_hbond utility. Number of hydrogen bond determined on the basis of donor-acceptor distance smaller than 3.6 Å and of donor-hydrogen-acceptor angle larger than 90°. SASA analysis formed in PKM2 was analyzed by using g_sas GROMACS, respectively. Distance between domains were calculated by using g_dist utility. To generate the plot for three-dimensional backbone RMSD, RMSF of carbon-alpha, gyration of backbone and SASA analysis, we used Graphing Advanced Computation and Exploration (GRACE) program.

### Cloning of wild and nsSVPs of PKM2 cDNA

Wild type PKM2 (PK-WT) cDNA was amplified using specific primers

Forward 5’ ATAT**GGATCC**ATGTCGAAGCCCCATAGTGA-3’

Reverse 5’-ATAT**CTCGAG**TCACGGCACAGGAACAACAC-3’ containing BamH1 and Xho-I restriction sites (bold letters), respectively; and sub-cloned in TA vector (In-vitrogen). For nsSVPs of PKM2 cDNA, following primers were used for site directed mutagenesis, replacing underlined nucleotide for 3 specific nsSVPs, identified as highly damaging by all prediction tools.

FP PKM2 C31F 5’-GAGCACATGTTCCGCCTGGAC-3’

RP PKM2 C31F 5’-GTCCAGGCGGAACATGTGCTC-3’

FP PKM2 Q310P 5’-TTCCTTGCTCCGAAGATGATG-3’

RP PKM2 Q310P 5’-CATCATCTTCGGAGCAAGGAA-3’

FP PKM2 S437Y 5’-TCTGGCAGGTATGCTCACCAG-3’

RP PKM2 S437Y 5’-CTGGTGAGCATACCTGCCAGA-3’

Once wild and nsSVPs cDNA were cloned in TA vector, they were digested with BamH1 and Xho-1 and sub-cloned in pGEX-6P2 vector. All the clones were confirmed by sequencing.

### Fusion protein expression, purification and estimation

pGEX-6P2 containing wild and nsSVPs of PKM2 were transformed in *E*. *Coli* strain BL21-DE3 by the heat shock method. Single colony was inoculated in LB medium overnight at 37°C under shaking conditions, followed by 1% inoculation of overnight grown culture in 200ml fresh LB. The culture was grown at 37°C for 2 hrs (till mid log phase) and induced with 1mM IPTG at 18°C for 12 hrs when culture was harvested by centrifugation and pellet stored at -80°C. For protein purification, the cell pellet was lysed in sonication buffer containing 50mM Tris-HCl (pH = 8), 500mM NaCl, 10% glycerol, 1mM PMSF and 1mM 2-mercaptoehtanol. The culture was lysed by an intermittent pulse of sonication and finally centrifuged at high speed to get a clear lysate. The supernatant was incubated with 400ul of GST beads (Amersham) for 4 hrs and beads washed 3–5 times, using 10 ml of sonication buffer. In order to obtain the GST tag free PKM2 protein, a fraction of washed beads was incubated with 80-U of Pre-Scission protease (GE Life sciences) at 5°C for 4 hours in prescribed conditions. Beads were then pelleted and the supernatant collected at 4°C. Protein quality was checked on running a fraction on 12% SDS PAGE and quantified using BCA protein estimation method (Pierce thermo scientific).

### Enzyme Activity assay

Enzymatic activity was assessed using a coupled reaction method as described earlier [6]. For a typical reaction a mixture containing 50mM Tris-pH 7.5, 6.75 mM MgCl_2_, 95mM KCl, 1.7mM PEP, 2.5 mM ADP, 0.281 mM NADH and 4.87 U of Lactate dehydrogenase was incubated with 0.2ug of purified protein for 5 minutes under real time OD measurement at 340 nM. A constant rate of drop in OD was noted for 5 minutes and activity in U/mg calculated using the equation:

**Activity (U/mg) =** Change in OD / 6.22 X mg of protein used per ml of reaction mixture X total time of reaction in minutes

Where, 6.22 mM/cm is the extinction coefficient for NADH.

Activity of wild and nsSVPs was calculated in the absence and presence of allosteric activator FBP (2mM) for wild type (PKWT) and nsSVPs. All experiments were carried out more than twice and Sigma Plot-10 was used to draw plots.

### Glycerol gradient analysis

GST tagged PKM2 C31F nsSVP was purified as described earlier [6]. 100 μg of purified protein was loaded on top of a 15–33% glycerol gradient (using 50 mM Tris-Cl buffer, pH 8, containing 150 mM NaCl, 1 mMphenylmethylsulfonyl fluoride) and centrifuged at 50,000 rpm for 16 h at 4°C, using an SW-55 Ti Beckman Coulter rotor. Fractions of 100 μl were taken starting from top of the gradient and assayed for PKM2 activity, which was dependent upon the proportionate representation of dimers and tetramers formed, as assessed routinely [6].

### Genotyping, statistical and computational analysis on intronic variations

High-throughput genotyping of SNPs was performed using IplexTM GOLD chemistry on a matrix-assisted laser desorption, ionization time-of-flight mass spectrometer (Sequenom). SNPs with a call rate <90% were removed. Significant SNPs had a call rate >95%. The individual call rate threshold was at least 95%. The statistical analysis of the polymorphic variant frequencies of candidate gene was performed with overall genotype and allele frequencies in a total of 388 patients and control subjects and compared between cases (205) and controls (183), using a 3 x 2 and 2 x 2 χ^2^ test. SPSS software, version 17 (SPSS), was used for statistical analysis. The genotype frequencies for SNPs were subjected to Hardy-Weinberg equilibrium (HWE) analysis in the combined samples (P <. 01) from HWE were removed as a quality control criterion. To analyze the influence of significant variants on transcription, we used the Human splicing factor tool, SpliceAid (http://www.introni.it/splicing.html), a most widely used software available to screen intronic SNPs, with default threshold levels. We conducted an *in silico* screen involving flanking nucleotides (10bps upstream and downstream) of these associated SNP alleles.

## Results

### *In-silico* analysis based on sequence and structure of nsSNPs and mutations in PKM2

**Fig 2:**
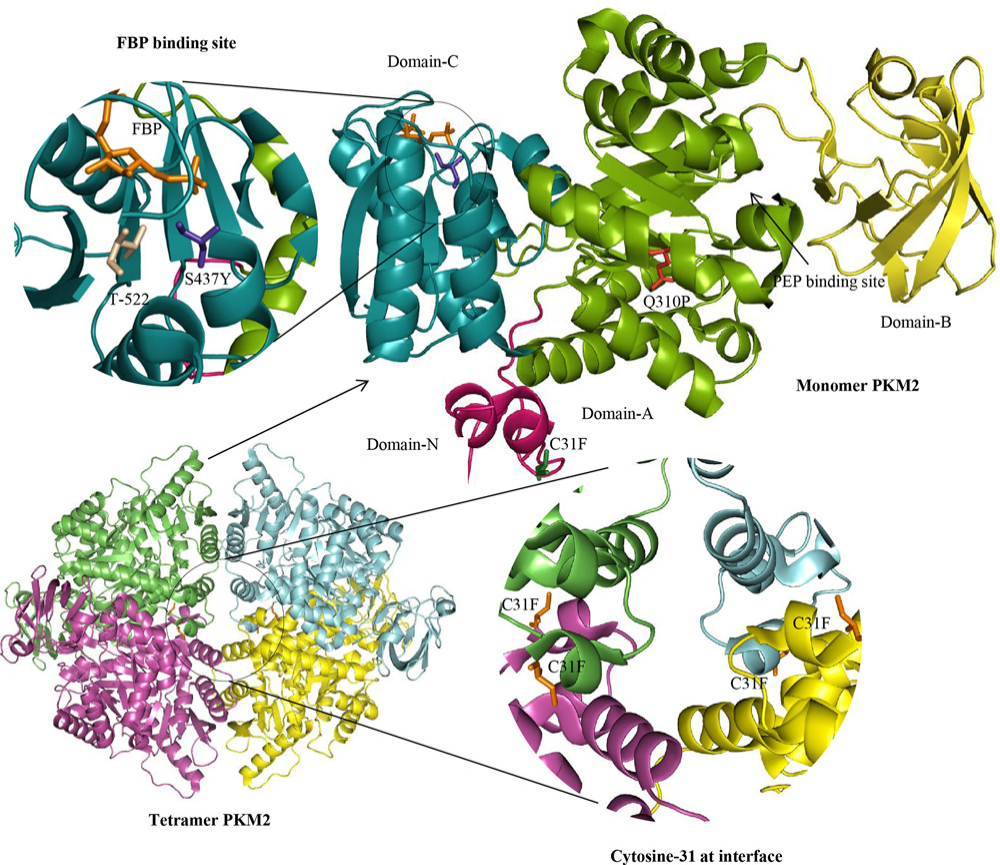
3D structure of wild PKM2 monomer. All three substitutions are highlighted in stick model, C31F in green color, Q310P in red color, S437Y in purple color and FBP highlighted in orange stick. Four domains of PKM2 are highlighted in the cartoon model with different colors.

The details of *in-silico* sequence and structure based analysis of 29 nsSNP and 2 mutations are provided in Tables 1 and 2; and the results of the combined analysis of SIFT, PolyPhen and PANTHER tools reflected in Table 1. Seven out of 31 nsSNPs were predicted as damaging by all these programs. SNP&GO, PhD-SNP, SNAP and SNPeffect tools predicted 9 nsSNPs as damaging (Table 2). We selected nsSNPs for further analysis which were predicted as damaging by at least 5 prediction programs with RI > 2 for SNAP and 3 for PhD-SNP and SNP&GO. The combined analysis of all tools predicted 5 nsSNPs as damaging in PKM2 (Table 3). Further structural analysis of 5 variant proteins (C31F, G204V, Q310P, R339P & S437Y) was carried out using HOPE (Have yOur Protein Explained) (http://www.cmbi.ru.nl/hope/home) [36], which predicted only 3 of these as highly damaging by all prediction tools. Q310P, located in an α-helix very close to the active site (Fig. 2), was predicted to disrupt the α-helix with replaced proline and affecting the structure of the protein severely (S1 Fig). S437Y was located in the part of the allosteric site, the binding site of FBP (Fig. 2). It was predicted that differences in amino acid properties could disturb this region and its function by causing the loss in interaction with FBP. It was also predicted that the variant tyrosine could loose hydrogen bond with threonine at position 522. C31F, located in the inter subunit contact domain (Fig. 2), was predicted to disturb the monomer or dimer interaction, by affecting the local stability, which in turn could affect the ligand contacts made by one of the neighboring residues. The 3 nsSNPs (C31F, Q310P & S437Y) chosen on the basis of HOPE (considering tetrameric PKM2) results were subjected to molecular dynamics simulations. The stability analysis of PKM2 tetramer using FOLDX predicted three of the nsSVPs (C31F, Q310P and S437Y) with significantly decreased stability in compared to the wild type (Table 4) is based on total energy. Though the entropy was not significant, the analysis has taken into consideration all the factors, including Van der Waals clashes, Electrostatics and Van der Waals, showing a significant total energy. A comparison of the dimeric form of wild and nsSVPs followed a similar pattern (S1 Table). Further, *in-vitro* protein activity of all the three nsSVPs were compared with the wild PKM2 (described later).

**Table 1:** Comparison of SIFT,PolyPhenand PANTHER Prediction.

dbSNP rs# clusterID	Protein residue	SIFT score	SIFT Prediction	HumDivScore	HumDiv Prediction	HumVarScore	HumVar Prediction	PolyPhen Prediction	PANTHER
rs11558365	Q16H	0.01	Affect protein function	0.075	Benign	0.022	Benign	Highly tolerant	-3.75
rs11558360	E28K	0.08	Tolerated	0.976	Probably damaging	0.691	Possibly damaging	Damaging	-3.51
rs11558375	C31F^a^	0.01	Affect protein function	0.999	Probably damaging	0.932	Probably damaging	Highly damaging	-4.18
rs147939689	R56Q	0.68	Tolerated	0.998	Probably damaging	0.74	Possibly damaging	Damaging	-2.96
rs142392595	Y83C	0	Affect protein function	1	Probably damaging	0.991	Probably damaging	Highly damaging	-2.90
rs146173648	A147T	0.39	Tolerated	0.023	Benign	0.013	Benign	Highly tolerant	-3.01
rs112954819	M149V	0.27	Tolerated	0	Benign	0.002	Benign	Highly tolerant	-3.54
rs61753428	N155S	0.5	Tolerated	0	Benign	0.001	Benign	Highly tolerant	-2.75
rs11558351	K186N	0.42	Tolerated	0.829	Possibly damaging	0.636	Possibly damaging	Slightly damaging	-3.58
rs11558354	G200C^a^	0	Affect protein function	1	Probably damaging	1	Probably damaging	Highly damaging	-6.22
rs141732747	S202F	0.71	Tolerated	0	Benign	0.002	Benign	Highly tolerant	-3.96
rs17853396	G204V^a^	0.04	Affect protein function	1	Probably damaging	0.999	Probably damaging	Highly damaging	-5.10
rs182730190	A214V	0.31	Tolerated	0.75	Possibly damaging	0.422	Benign	Tolerant	-3.25
rs143294717	S222L^a^	0	Affect protein function	0.799	Possibly damaging	0.482	Possibly damaging	Slightly damaging	-6.31
rs149108298	E234K	0.54	Tolerated	0.999	Probably damaging	0.98	Probably damaging	Highly damaging	-2.47
rs149152236	Q235L	0.02	Affect protein function	0.325	Benign	0.248	Benign	Highly tolerant	-3.83
rs145060432	K266R	0.15	Tolerated	0.028	Benign	0.066	Benign	Highly tolerant	-4.44
rs147032160	R278Q	0.9	Tolerated	0.277	Benign	0.079	Benign	Highly tolerant	-3.19
rs11558370	Q310P^a^	0	Affect protein function	1	Probably damaging	1	Probably damaging	Highly damaging	-7.17
rs147956260	R316W	0	Affect protein function	0.675	Possibly damaging	0.355	Benign	Tolerant	-4.87
rs149836418	N318S	0	Affect protein function	0.04	Benign	0.064	Benign	Highly tolerant	-4.82
rs2959910	R339P	0	Affect protein function	0.423	Benign	0.29	Benign	Highly tolerant	-5.94
rs151078084	Q378E^a^	0.01	Affect protein function	0.999	Probably damaging	0.982	Probably damaging	Highly damaging	-4.65
	H391Y*	0.63	Tolerated	1	Probably damaging	0.995	Probably damaging	Highly damaging	-3.19
rs181813553	A402V	0.18	Tolerated	0	Benign	0	Benign	Highly tolerant	-2.91
rs148035865	P408L	0.36	Tolerated	0.002	Benign	0.009	Benign	Highly tolerant	-3.63
	K422R*	0.05	Tolerated	0.002	Benign	0.032	Benign	Highly tolerant	-3.38
rs139159354	R436T	0.27	Tolerated	0.742	Possibly damaging	0.474	Possibly damaging	Slightly damaging	-3.18
rs59430203	S437Y^a^	0	Affect protein function	0.922	Possibly damaging	0.814	Possibly damaging	Slightly damaging	-4.12
rs141505399	R461H	0	Affect protein function	0.498	Possibly damaging	0.18	Benign	Tolerant	-0.12
rs11558358	V490L	0.34	Tolerated	0.8	Possibly damaging	0.393	Benign	Tolerant	-0.12

^a^ Pyruvate kinase M2 nsSNPs predicted as damaging by both SIFT,PolyPhen and PANTHER.

* known mutations of human pyruvate kinase M2.

**Table 2:** Prediction based on SVM, Neural Network method and SNPeffect.

Protein residue	SNP-GO	RI SNP-GO	PhD-SNP	RI PhD-SNP	SNAP	RI SNAP	FoldX	TANGO	WALTZ	LIMBO
Q16H	Neutral	1	Disease	1	Non-neutral	1	Reduces	Not affect	Not affect	Not affect
E28K^a^	Disease	4	Disease	6	Non-neutral	0	Slightly Reduces	Not affect	Not affect	Not affect
C31F^a^	Disease	6	Disease	9	Non-neutral	3	Reduces	Not affect	Not affect	increases
R56Q	Disease	1	Disease	3	Neutral	5	Slightly Reduces	Not affect	Not affect	Not affect
Y83C	Disease	5	Disease	2	Neutral	2	Slightly Reduces	Not affect	Not affect	Not affect
A147T	Neutral	4	Disease	1	Neutral	6	No effect	Not affect	Not affect	Not affect
M149V	Neutral	5	Neutral	4	Neutral	4	Reduces	Not affect	Not affect	Not affect
N155S	Neutral	6	Neutral	1	Neutral	6	No effect	Not affect	Not affect	Not affect
K186N	Disease	0	Disease	4	Neutral	5	Reduces	Not affect	Not affect	Not affect
G200C^a^	Disease	7	Disease	5	Non-neutral	1	Reduces	Not affect	Not affect	Not affect
S202F	Disease	0	Disease	3	Neutral	7	No effect	Not affect	Not affect	Not affect
G204V	Disease	5	Neutral	0	Non-neutral	3	Reduces	Not affect	Not affect	Not affect
A214V	Neutral	8	Neutral	9	Neutral	3	Enhances	Not affect	Not affect	Not affect
S222L	Disease	8	Disease	2	Non-neutral	0	Slightly Enhances	Not affect	Not affect	Not affect
E234K	Disease	0	Neutral	2	Neutral	5	No effect	Not affect	Not affect	Not affect
Q235L	Disease	2	Neutral	1	Neutral	1	Enhances	Not affect	Not affect	Not affect
K266R	Neutral	8	Neutral	5	Neutral	6	No effect	Not affect	Not affect	Not affect
R278Q	Neutral	4	Neutral	1	Neutral	8	No effect	Not affect	Not affect	Not affect
Q310P^a^	Disease	9	Disease	7	Non-neutral	5	severely reduces	Not affect	Not affect	Not affect
R316W^a^	Disease	5	Disease	8	Non-neutral	1	Reduces	Not affect	Not affect	Not affect
N318S	Disease	7	Disease	8	Neutral	2	Reduces	Not affect	Not affect	Not affect
R339P^a^	Disease	8	Disease	8	Non-neutral	4	Reduces	Not affect	Not affect	Not affect
Q378E^a^	Disease	7	Disease	7	Non-neutral	0	Reduces	Not affect	Decreases	Not affect
H391Y*	Neutral	7	Neutral	1	Neutral	4	Reduces	Increases	Increases	Not affect
A402V	Neutral	8	Neutral	7	Neutral	5	Slightly Reduces	Not affect	Not affect	Not affect
P408L	Neutral	1	Disease	4	Neutral	2	Reduces	Not affect	Not affect	Not affect
K422R*	Neutral	3	Neutral	0	Neutral	6	No effect	Not affect	Not affect	Not affect
R436T	Disease	2	Disease	1	Neutral	3	No effect	Not affect	Not affect	Not affect
S437Y^a^	Disease	7	Disease	4	Non-neutral	3	Reduces	Not affect	Not affect	Not affect
R461H^a^	Disease	7	Disease	9	Non-neutral	0	Reduces	Not affect	Not affect	Not affect
V490L	Neutral	3	Neutral	5	Neutral	7	no effect	Not affect	Not affect	Not affect

**Table 3:** Functionally important nsSNPs predicted by *in-silico* tools.

dbSNP rs# clusterID	Protein residue	SIFT Prediction	PolyPhen Prediction	PANTHER	SNP-GO	PhD-SNP	SNAP	FOLDX
rs11558375	C31F	Affect protein function	Highly damaging	-4.18	Disease	Disease	Non-neutral	Reduces
Rs17853396	G204V	Affect protein function	Highly damaging	-5.1	Disease	Disease	neutral	Reduces
rs11558370	Q310P	Affect protein function	Highly damaging	-7.17	Disease	Disease	Non-neutral	Severely Reduces
Rs2959910	R339P	Affect protein function	Highly tolerant	-5.94	Disease	Disease	Non-neutral	Reduces
rs59430203	S437Y	Affect protein function	Slightly damaging	-4.12	Disease	Disease	Non-neutral	Reduces

**Table 4:** Tetramer PKM2 stability analayis of functionally important nsSNPs.

Energy	S437Y	C31F	Q310P	G204V	R339P	WILD
Total energy	-169.18	-201.784	-234.829	-261.849	-271.499	-284.002
Backbone Hbond	-1588.78	-1598.37	-1590.27	-1605.78	-1589.39	-1606.71
Sidechain Hbond	-705.686	-712.471	-694.925	-712.105	-679.437	-706.276
Van der Waals	-2618.94	-2613.89	-2614.9	-2629.8	-2613.46	-2623.44
Electrostatics	-165.318	-153.948	-145.856	-156.888	-152.165	-147.729
Solvation Polar	3584.64	3564.06	3568.62	3595.31	3557.18	3587.1
Solvation Hydrophobic	-3435.62	-3434.93	-3440.93	-3457.38	-3440.49	-3450.97
Van der Waals clashes	97.0956	76.6626	70.4648	65.8795	64.6183	61.9747
entropy sidechain	1390.36	1391.43	1383.19	1396.09	1367.1	1393.05
entropy mainchain	3301.63	3303.96	3266.44	3277.26	3255.39	3247.34
torsional clash	24.1403	24.36	21.7288	24.722	17.7714	20.4669
backbone clash	1419.92	1420.51	1424.05	1434.01	1429.6	1433.56
helix dipole	-49.5064	-48.7697	-56.5457	-56.6833	-56.381	-55.4927
electrostatic kon	-7.32959	-7.17923	-7.14662	-7.73646	-6.35098	-7.32937
energy Ionisation	4.13314	5.41619	5.31339	5.27333	4.11382	4.01702
Number of Residues	2072	2072	2072	2072	2072	2072

### Molecular dynamics simulations

**Fig 3:**
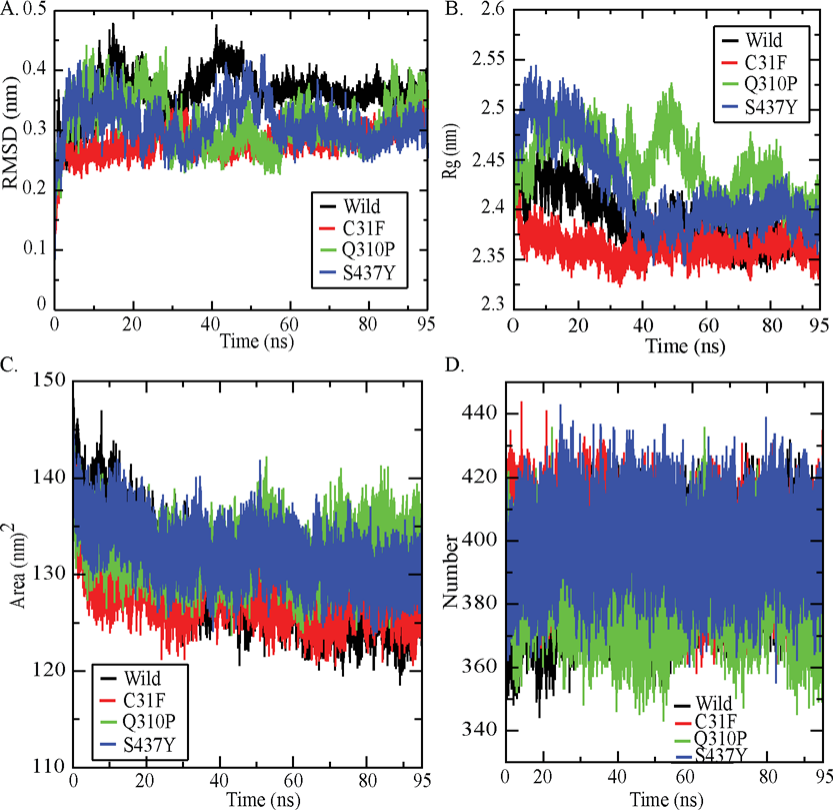
PKM2 structural properties of wild and nsSVPs. (A) Backbone RMSD of PKM2 (B) Radius of gyration of protein (C) Solvent accessible surface area (D) intra-molecular hydrogen bonds.

**Fig 4:**
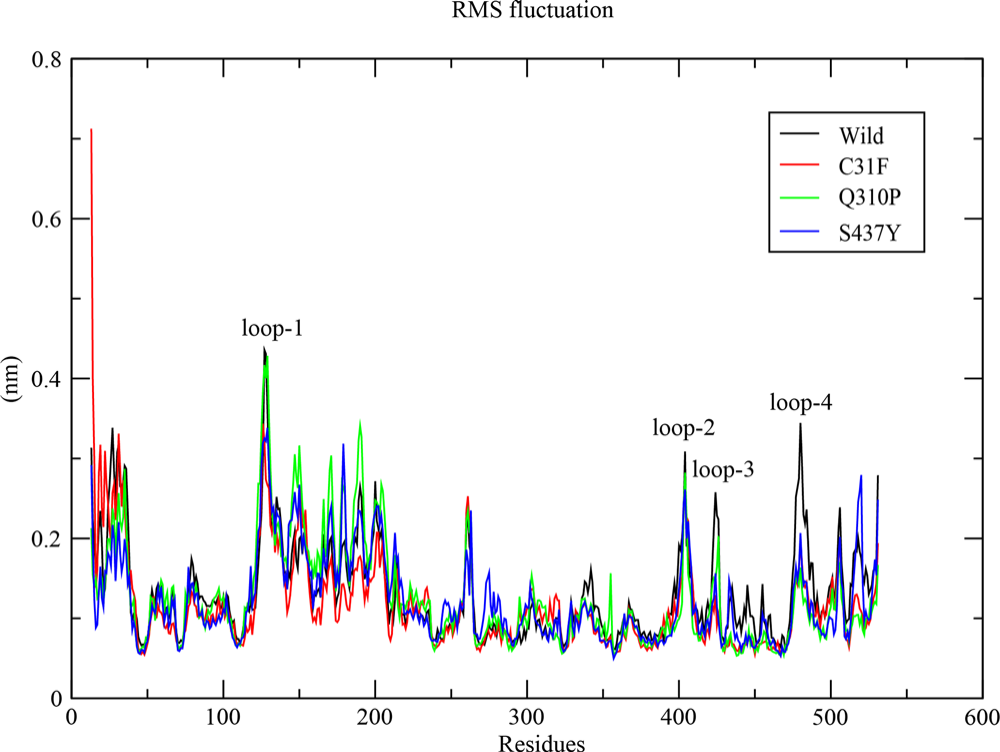
PKM2 Root mean square fluctuation of wild and nsSVPs. The C-alpha RMF of wild type PKM2 (black), C31F (red), Q310P (green) and S437Y (blue).

**Fig 5:**
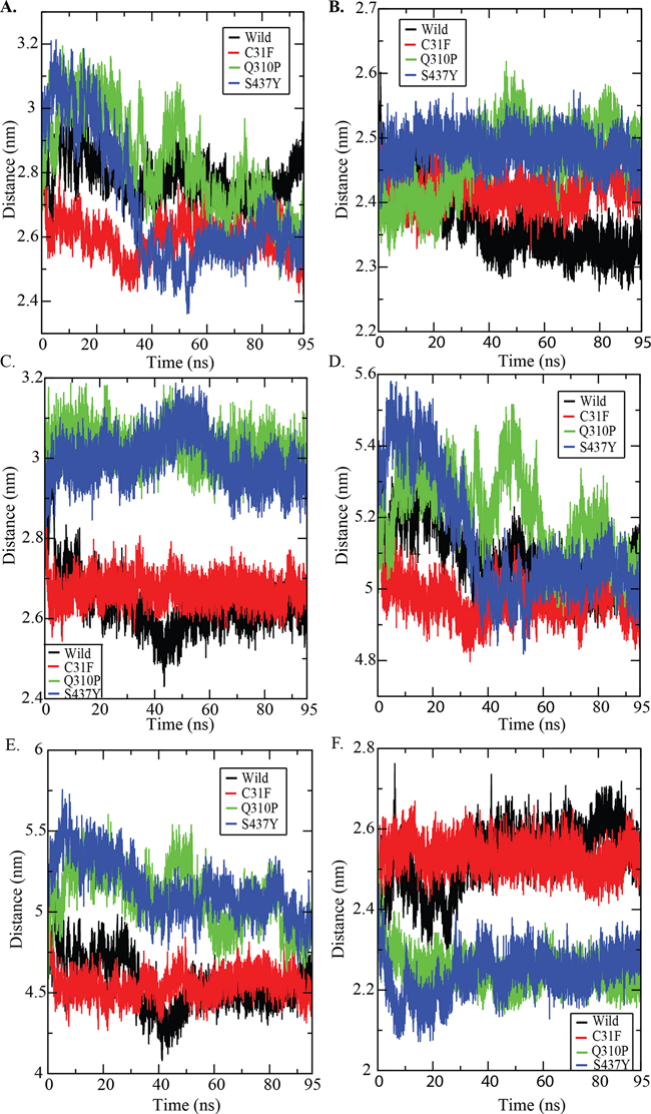
Distance between each domain of PKM2 with function of time. (A) Distance between domain A & B (B) Distance between domain A & C (C) Distance between domain A & N (D) Distance between domain B & C (E) Distance between domain B & N (F) Distance between domain C & N. The wild type PKM2 highlighted in black and nsSVPs C31F, Q310P and S437Y were highlighted in red, green and blue, respectively.

We performed molecular dynamics simulations for wild type and other three substitutions (C31F, Q30P and S437Y). The average value of RMSD for backbone atoms was 0.363 nm in wild type PKM2. Whereas, the average values of RMSD for backbone atoms in C31F, Q310P and S437Y were 0.282, 0.314 and 0.312, respectively (Fig. 3A). RMSD of native and nsSVPs as a function of time were within 0.5Å +/- during the simulation, which suggested that the simulation is stable for further structural comparison. The radius of gyration (Rg) value of protein for wild varied between 2.336 nm to 2.484 nm. For C31F, Q310P and S437Y, the Rg value varied between 2.322 nm to 2.463 nm, 2.365 nm to 2.527 nm and 2.343 nm to 2.545 nm, respectively (Fig. 3B). The average values of radius of gyration (Rg-protein) for wild and 3 variant PKM2 proteins were 2.390 nm, 2.364 nm, 2.445 nm and 2.422 nm, respectively. The lower values of radius of gyration in wild protein structure suggested it to be stable. These data revealed that the stability of the PKM2 decreased upon two (Q310P and S437Y) substitutions; and in case of C31F variant protein, it did not show a significant decrease in stability when compared to wild PKM2 (Fig. 3B). However, when stability analysis in tetrameric PKM2 structure using FOLDX (based on total energy) was carried out, it showed a significant difference in stability between the wild and variant C31F PKM2. Further, glycerol gradient results of the C31F-PKM2 protein showed more of the relatively inactive dimeric form as compared to functionally active tetramer, apparently supporting that C31F nsSVP is less stable (S2 Fig). Solvent accessible surface area (SASA) was calculated for wild type and variant proteins trajectories (Fig. 3C) for each residue [37] and values averaged. The average SASA of wild type and nsSVPs were: 129.768 nm^2^, 128.339 nm^2^, 132.527 nm^2^ and 132.161 nm^2^, respectively. The SASA of wild type PKM2 varied from 118.497 nm^2^ to 148.950 nm^2^; and that of variant proteins C31F, Q310P and S437Y, differed from 120.376 nm^2^ to 147.990 nm^2^, 123.296 nm^2^ to 147.333 nm^2^ and 123.634 nm^2^ to 1487.204 nm^2^, respectively. The number of intra-molecular hydrogen bonds were calculated for wild and variant proteins to assess the fluctuation of the rigidity of the proteins. The wild type and three nsSVPs, C31F, Q310P and S437Y, showed 389, 397, 386 and 399, intra-molecular hydrogen bonds, respectively (Fig. 3D). The RMSF differences indicated that the dynamics of the wild and the 3 variants had four loop regions that displayed larger flexibility. One of the loops (the loop-1) was located at the PEP binding site and the rest of the 3 were near FBP binding site. This fluctuation in the structure allowed us to calculate the distance between each domain of PKM2 (Fig. 4). The distance between each domain was calculated to identify the effect of these variations in the domains of PKM2; where the distance between the domains A and B decreased significantly in the case of nsSVPs when compared to wild type PKM2 (Fig. 5A). Incidentally, the binding pocket of the substrate (PEP) was located in-between domains A and B (Fig. 2). The nsSVPs of PKM2 showed a slightly higher distance between the domains A and C, when compared to wild type PKM2 (Fig. 5B). The wild type and variant C31F followed the same pattern in the domain distance between A and N, showing larger distance comparable to other two, Q310P and S437Y, variant proteins which followed the same pattern (Fig. 5C). The S437Y variant protein showed a sudden decrease in the distance between domain B and C between 10ns to 40ns; and after 40ns the distance was stable (Fig. 5D). The distance between the domains A and N was reflected in-between domains, B and N, as well (Fig. 5E). Whereas, a larger distance between domains C and N was observed in wild type and C31F variant, when compared to Q310P and S437Y nsSVPs (Fig. 5F).

### PKM2 Activity Assay

**Fig 6:**
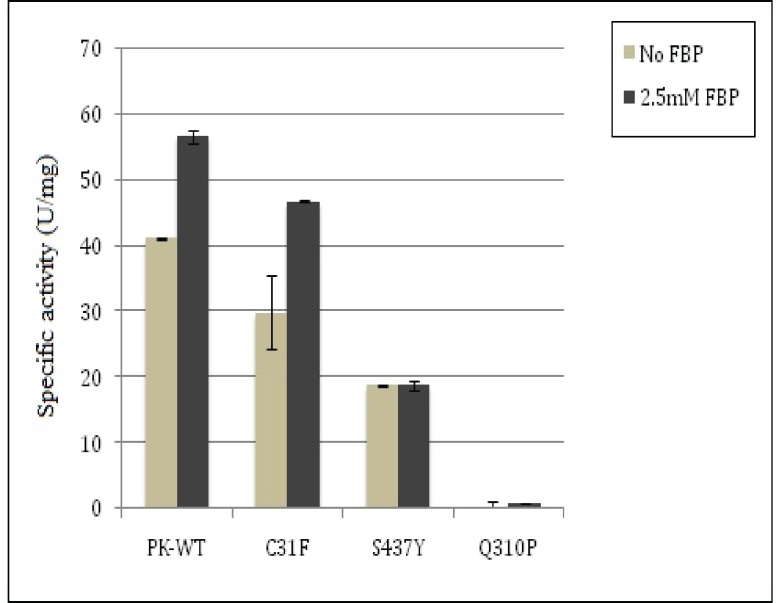
Enzyme activity of wild and variants. Under optimal conditions, the activity assay of purified PK-WT and variant proteins showed ~18% and 55% reduction in C31F and S437Y nsSVPs activities respectively. However, Q310P nsSVP was catalytically dead. Binding of allosteric activator FBP increased the activity up-to 27% and 35% in PKWT and C31F nsSVP, however, showed no increase in activity in S43Y nsSVP.

The purified recombinant wild type human-PKM2 protein (PKWT) and its nsSVPs expressed in *E*. *coli* was used for PKM2 activity assay. PKWT showed ~41.1 U/mg of PKM2 activity while C31F and S437Y variants showed 29.9 and 18.82 U/mg activity, respectively (Fig. 6). Interestingly, another non-synonymous variant, Q310P, was catalytically dead (Fig. 2). Since PKM2 allosteric activator FBP is known to increase its affinity for substrate PEP with a net increase in activity [6], in order to investigate the effect of non-synonymous variations on FBP dependent change in activity, we incubated proteins with 2mM FBP and measured the activity under the similar optimal condition as assessed in the absence of FBP. As expected, FBP increased the activity by 27% in the case of wild PKM2. In C31F variant, a 35% increase in activity in the presence of FBP was significantly higher than wild type which is not expected. The S437Y variant did not show any change in activity, while Q310P variant remained catalytically dead relatively. All these results indicated the possible change in the structures of variants which potentially led to modulations in binding of either substrate or other ligands like FBP, ADP to the enzyme.

### Genotyping and *in-silico* functional analysis of SNPs associated with sporadic breast cancer

**Fig 7:**
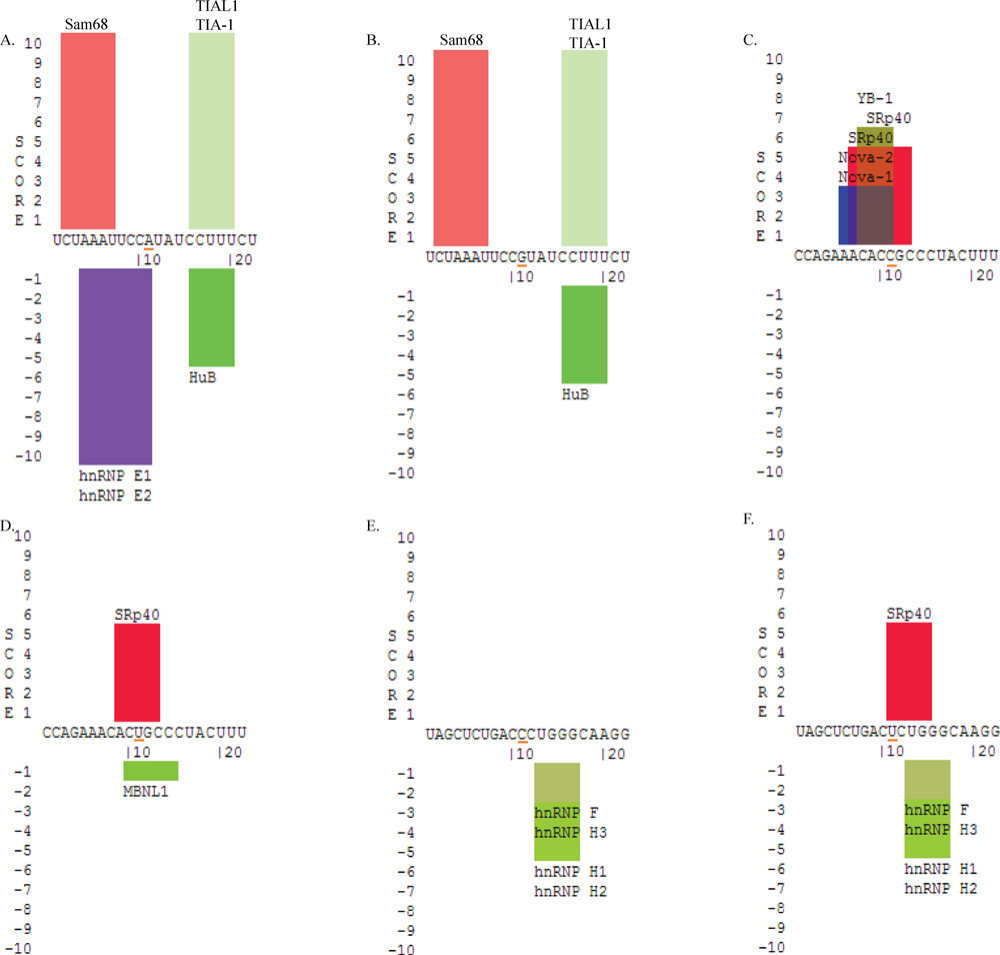
Prediction of Splice regulatory proteins to the PKM2 gene. (A) Splicing factor binding in presence of rs2856929_A (B) splicing factor binding in presence of rs2856929_C (C) splicing factor binding in presence of rs8192381_C (D) splicing factor binding in presence of rs8192381_U (E) splicing factor binding in presence of rs8192431_C (F) splicing factor binding in presence of rs8192431_U.

A population based case-control study carried out on 18 SNPs within PKM2 gene, including 15 intronic and 3 in the neighbouring UTR regions (2 close to 5’UTR and 1 close 3’UTR) in 205 sporadic breast cancer cases and 183 controls from northern India (Table 5), showed only 5 intronic SNPs (rs8192386, 0.029; rs1037680, 0.043; rs8192431, 0.053; rs2856929, 0.062; rs8192381, 0.088) in strong association with breast cancer (Table 5). Out of the 5 SNPs, 3 SNPs (rs8192386, rs1037680 and rs8192381) was located in intron 1; whereas other two SNPs (rs2856929 and rs8192431) were located in intron 8 and 10, respectively. To identify the role of these SNPs *in silico*, SpliceAid2 predicted the splicing factor binding to the minor and major alleles of these SNPs and its flanking sequences. Thus, multiple putative splice regulatory proteins binding sites were identified that could modulate PKM2 pre-mRNA splicing. Due to the single nucleotide difference in the sequences, representing minor and major alleles, 3 (rs8192431, rs2856929 and rs8192381) out of 5 SNPs, were predicted to influence the binding of different splicing factors (Fig. 7). SpliceAid2 identified rs2856929_A allele binding to hnRNP E1 and hnRNP E2 (Fig. 7A), whereas in case of rs2856929_G allele, the binding of these splicing factors was inhibited (Fig. 7B). Another SNP, rs8192381_C, allowed the binding of YB-1, SRp40, Nova-1 and Nova-2 (Fig. 7C); whereas rs8192381_U allele inhibited the binding of these four splicing factors, but allowed the binding of MBNL1 (Fig. 7D). SNP, rs8192431_C, inhibited the binding of SRp40 splicing factor (Fig. 7E), and in case of rs8192431_U the binding was possible (Fig. 7F).

**Table 5:** Allele and genotype frequencies for 5 significant SNPs within PKM2.

SNP ID	Region	Chi P Value	Allelic P Value
rs8192386_C/A*	Intron	0.094	0.030
rs1037680_T/C*	Intron	0.127	0.044
rs8192431_C/T*	Intron	0.152	0.053
rs2856929_A/G*	Intron	0.158	0.062
rs8192381_C/T*	Intron	0.170	0.089
rs8040828_A/C	Intron	0.369	0.266
rs4506844_T/C	Intron	0.509	0.276
rs8192425_A/G	Intron	0.310	0.467
rs3743227_C/G	5’ near	0.698	0.701
rs141487927_G/G	Intron	Homozygous	
rs143577436_G/G	Intron	Homozygous	
rs186844011_G/G	Intron	Homozygous	
rs916133_T/T	Intron	Homozygous	
rs184167700_G/G	Intron	Homozygous	
rs2445734_G/G	Intron	Homozygous	
rs2607089_G/G	Intron	Homozygous	
rs12443060_T/T	3’ near	Homozygous	
rs8192347_C/C	5’ near	Homozygous	

*Five significant SNPs

## Discussion

PKM2 in the recent past has been assigned the role of a protein kinase [38]; and its multifunctional role, some proven and yet others speculative [9], have suggested it to be a co-activator, a transcription factor [17]. PKM2, thus, attains importance as a molecule within a cell to be studied in all perspectives. Lack of information about its functional status, in the background of natural polymorphisms within PKM2 suggests to initiate data-mining of such variations and mutations within the gene, followed by their *in silico* and functional characterization. Not much is known apart from the two known PKM2 gene mutations, reported earlier in Bloom syndrome cells [5], characterized for their possible biological function [6,8,9]. It was, therefore, essential to screen and identify highly damaging nsSNPs which could significantly affect the function of PKM2 using different prediction programs. The 29 nsSNPs and two mutations chosen for sequence analysis, 20 substitutions (64.5%) were predicted as damaging, although PANTHER predicted 77.4% of the substitutions as deleterious. Through structural analysis, using SNPeffect and HOPE, only three substitutions (C31F, Q310P and S437Y) within ISCD region of the protein, near PEP binding site and FBP binding pocket, respectively, were predicted as common between all the programs used. The nsSVPs predicted to affect the function in some programs were not reflected in other programs, assumingly due to the differences in the parameters of prediction in different algorithms; which illustrated the need of using more than one algorithm to obtain greater reliability in such predictions. Here we did not use a single method; and the consensus results were used for further analysis. Since the accuracies of the prediction tools (SNAP, SNPs&GO, SIFT, PolyPhen-2, PhD-SNP) are estimated to be between 50% and 80% [25,39], we filtered the common mutations from all the programs and decided to carry out MD simulations and *in vitro* experiments to validate the predictions.

Non-synonymous variant, C31F, located in the inter subunit contact domain (ISCD, a protein monomer interface where it comes in contact with another monomer to become an oligomeric protein) showed ~27% reduced activity in comparison to the wild type-non-variant PKM2 (PKWT). Despite the location of ISCD region positioned far away from the substrate or allosteric activator binding sites [4], the variation played an important role in transferring signals from one substrate binding site to another in the allosteric protein. Communicating from one substrate binding site to the other increases their net activity, depicted as a typical sigmoidal kinetics. A change in single amino acid could result in a local to distant structural change, leading to a variety of alterations in protein catalytic activity and behavior [6,7]. The studied variations within PKM2 led us to a similar conclusion when we co-related our *in silico* and *in vitro* studies. In the presence of FBP, C31F, showed a 35% increase in activity this also reflected in the MD simulation. Non-synonymous variations at critical positions within ISCD region do have a potential to affect protein oligomerisation [6,7], which could be a cause of reduction in protein activity in case of C31F-PKM2 variant, an area of interest for further investigation. The tetramer structural comparison of wild and C31F nsSVP using PDBsum depicted the structural change in tetramer form of C31F nsSVP when compared to the wild type and other nsSVPs (S3 Fig). This proved that C31F could have a major impact in the ISCD region of PKM2. The experimental results of C31F nsSVP of PKM2 showed the preferential presence of dimeric form. However, in presence of FBP (data not shown) there was a relative increase in the tetramer peak.

Further, non-synonymous variations in non-ISCD region also had their impact on protein function. In case of Q310P variation, located in an α-helix closer to the PEP binding site, replacement of a glutamine with proline could disrupt the α-helix and affect binding of PEP to the protein, rendering the variant catalytically inactive. This was proven in our activity assay, where the Q310P-PKM2 variant protein was observed to be catalytically dead, which was also predicted as the most damaging by prediction tools. Even upon binding with FBP, the net increase in activity was not significant in comparison to the wild and other variant proteins (Fig. 6), indicating that the variation probably did not affect FBP binding. Whereas PEP binding was severely affected, leading to no significant change in activity, despite FBP binding. The nsSNP, Q310P, affected the formation of 3D structure, which incidentally was not reflected in MD simulation. This observation clearly reflected on the local but significant alteration in protein structure. The *in silico-in vitro* correlation for FBP binding to PKM2 could also be confirmed by another example of S437Y variant. Since S437Y is located in the FBP binding site of the PKM2 this is expected that in the presence of FBP there would be no net change in PKM2 activity (Fig. 6). Replacing serine 437 with tyrosine may have affected FBP binding to proteins. However, the variant showed a 35% reduction in activity, even in the absence of FBP in comparison to PKWT, probably suggesting that a local structural change could be transmitted globally in a dynamic protein. Particular S437Y mutation is well supported by the Eyal Gottlieb’s group, where they also identified S437Y mutation influencing the binding of FBP in PKM2 [40]. RMSF analysis using MD simulation trajectories showed that residues 128 and 180–183 were influenced by S437Y nsSVP (Fig. 4). When a S437Y nsSVP crystal structure (PDB ID: 3G2G) was compared with *in-silico* mutated S437Y structure with an RMSD of 0.4Å deviation, it showed that both the structures were similar. The complex of FBP and L-serine with S437Y nsSVP showed 11 hydrogen bonds and 2 hydrophobic contacts, respectively (S4 Fig). In case of wild type PKM2 FBP and L-serine showed 15 hydrogen bonds and 3 hydrophobic contacts, respectively (S4 Fig). We have already shown in a previous study how a mutation resulting in an amino acid change from histidine to tyrosine could have a local to global structural change in PKM2 protein, affecting protein mobility, binding to different ligands and its functions [6,7]. We found that this may happen because of the predicted formation of a new hydrogen bond in the local hinges of protein, restricting its mobility and controlling its allosteric behavior [6]. In case of S437Y variant, HOPE also predicted a loss of hydrogen bond with threonine at position 522 replaced by tyrosine, which could give rise to all the changes described.

Further, to unravel the role of functionally important intronic and neighbouring-UTR SNPs in PKM2, the study also genotyped the known SNPs in these regions of the gene to detect the variants associated with sporadic breast cancer in Indian population, not reported previously in Indian population. Genotyping and statistical analysis identified 5 SNPs shows an association with sporadic breast cancer; however, only 3 SNPs could predict a functional role by influencing the binding of the splicing factor. The SNP (rs8192431) influenced the SRp40, which is known to be highly expressed in breast cancer. It is also reported that the higher expression of SRp40 correlates with alternative pre-mRNA splicing of CD44 [41]; suggesting the possibility of the SNP, rs8192431, to be used as a marker for breast cancer. A strong association observed of the breast cancer with the SNP, rs2856929, influenced the binding of HnRNP E1 and E2; where HnRNP proteins have been reported to deregulate pyruvate kinase mRNA splicing in cancer [42]. Altogether, the three intronic SNPs (rs8192431, rs2856929 and rs8192381) reflected a potential to alter the splicing mechanism of PKM2 gene, which could result in its aberrant expression and activity in cancer. This *in silico* observation provides a lead for future studies to understand how an aberrant expression and activity of PKM2, as a result of intronic-genetic variations, besides the known post-translational modifications (phosphorylation, oxidation, acetylation or hydroxylation), involved in inactivating the enzyme could result in inducing pro-cancerous metabolic alterations in cells [17,43,44]. These alterations in PKM2 activity in cancer conditions are at times due to subunit dissociation [43], or because of very rare dominant negative mutations [6,7]. In either of these conditions, PKM2 shows a reduced activity and a tendency to favour pro-cancerous features in a cell [8,9].

## Conclusion

This study integrates computational, genetic and experimental approaches to identify and analyze SNPs and mutations in PKM2 gene, considered as a metabolic tuner in cancer cells [8,9,45]. Crucial non-synonymous and intronic variations in PKM2 gene, affecting its activity, allosteric property and splicing, suggest their importance in the biological processes, since PKM2 has been implicated in cancer biology. The three nsSNPs affected PKM2 in three different ways, C31F could affect the oligomerization of PKM2; Q310P, affected the activity completely; S437Y, affected its allosteric nature. Interestingly, 3 intronic polymorphisms of functional relevance on the basis of *in-silico* studies, with a potential of generating splice variants and aberrant PKM2 protein, could be evaluated as cancer markers, and their prevalence in cancer patients and general population assessed for the proposed metabolic relevance.

## Supporting Information

S1 Fig HOPE result for Q310P. It breaks the helix which is in proximity to the PEP binding site; because of proline one hydrogen bond is missing which is highlighted by the arrow.
(TIF)

S2 Fig Glycerol gradient experiment of C31F nsSVP. The gradient showed a prominent dimer peak (fraction no.8–23) and a small tetramer peak (fraction no. 23–30) (Details in Materials and Methods).
(TIF)

S3 Fig Tetrameric PKM2 structural interface analysis. (A) Interface interaction in wild type PKM2 (B) Interface interaction in C31F nsSVP of PKM2 (C) Interface interaction in Q310P nsSVP of PKM2 (D) Interface interaction in S437Y nsSVP of PKM2.
(TIF)

S4 Fig Ligplot result of FBP and L-Serine. (A) Interaction between wild type PKM2 and FBP (B) Interaction between wild type PKM2 and L-Serine (C) Interaction between S437Y nsSVP and FBP (D) Interaction between S437Y nsSVP and L-Serine.
(TIF)

S1 Table Dimeric PKM2 stability analayis of functionally important nsSNPs. (DOCX)
