# Supplementary figures and images for: In Silico Screening, Genotyping, Molecular Dynamics Simulation and Activity Studies of SNPs in Pyruvate Kinase M2
Source: PLoS One. 2015 Mar 13;10(3):e0120469. doi: 10.1371/journal.pone.0120469 (PMC4359060; doi:10.1371/journal.pone.0120469)

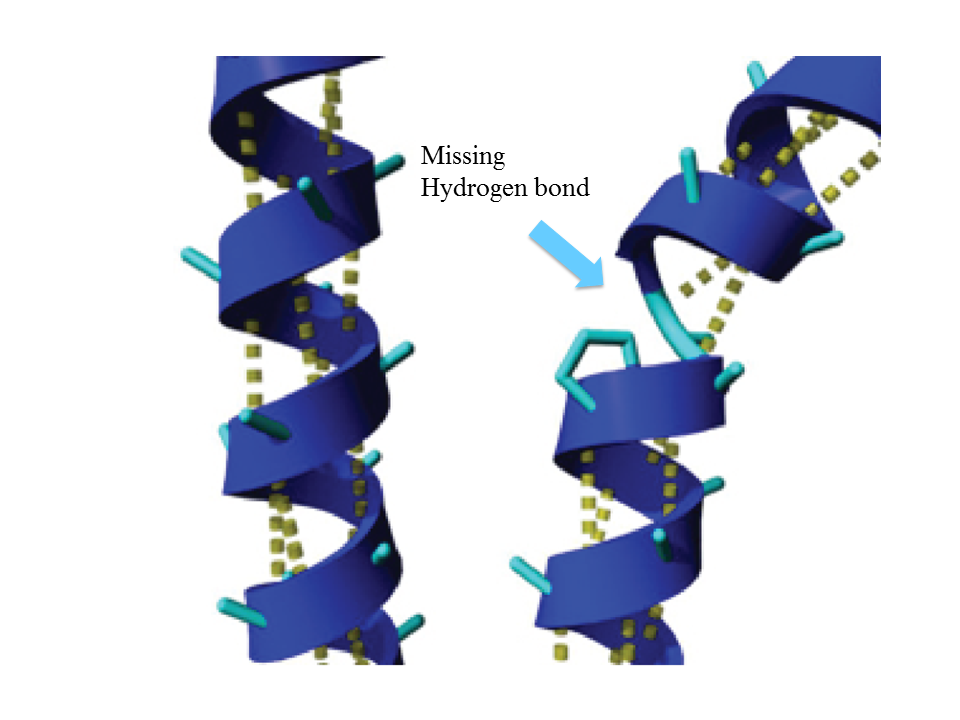

Supplement: S1 Fig — It breaks the helix which is in proximity to the PEP binding site; because of proline one hydrogen bond is missing which is highlighted by the arrow. (TIF) [file pone.0120469.s001.tif]

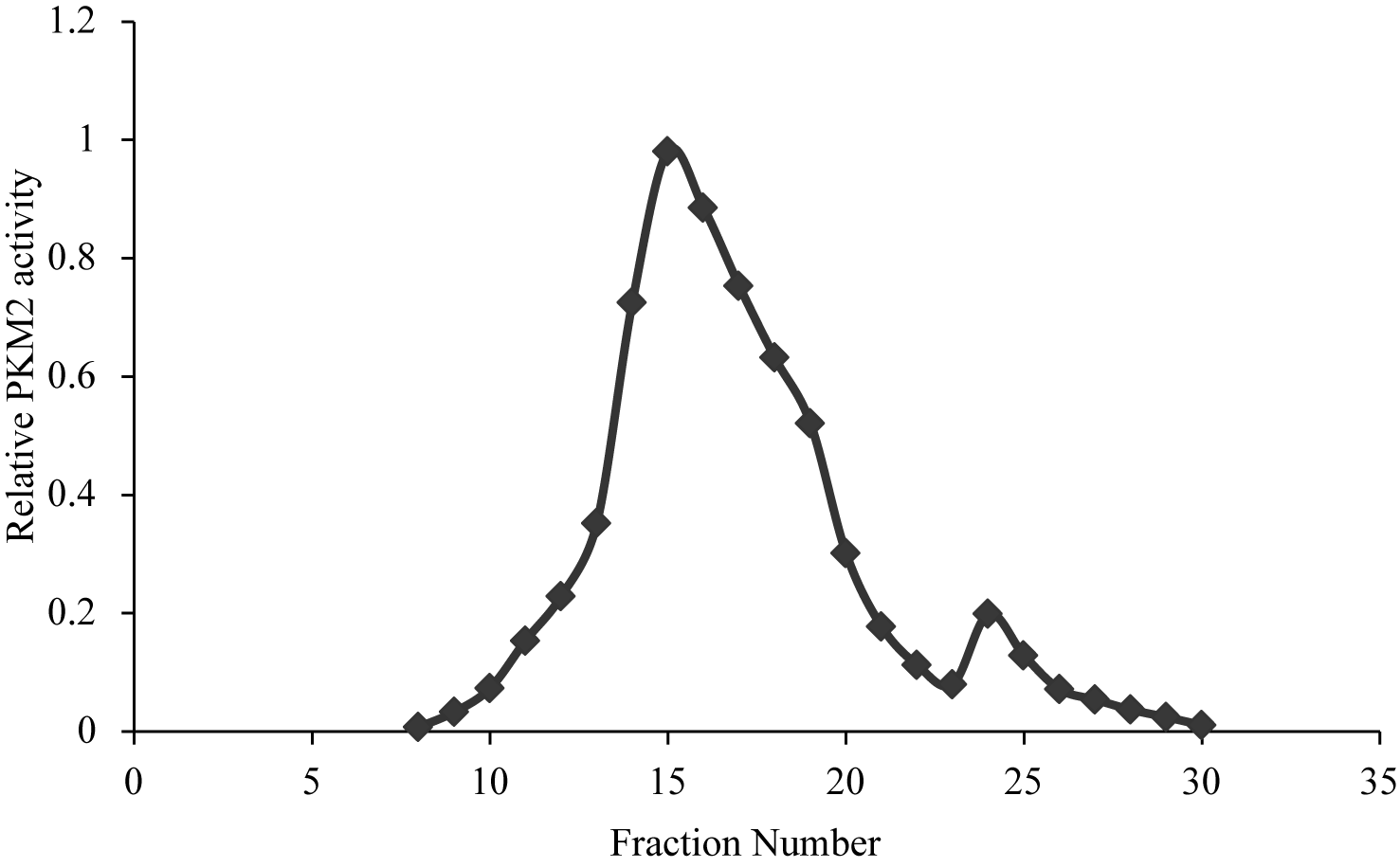

Supplement: S2 Fig — The gradient showed a prominent dimer peak (fraction no.8–23) and a small tetramer peak (fraction no. 23–30) (Details in Materials and Methods). (TIF) [file pone.0120469.s002.tif]

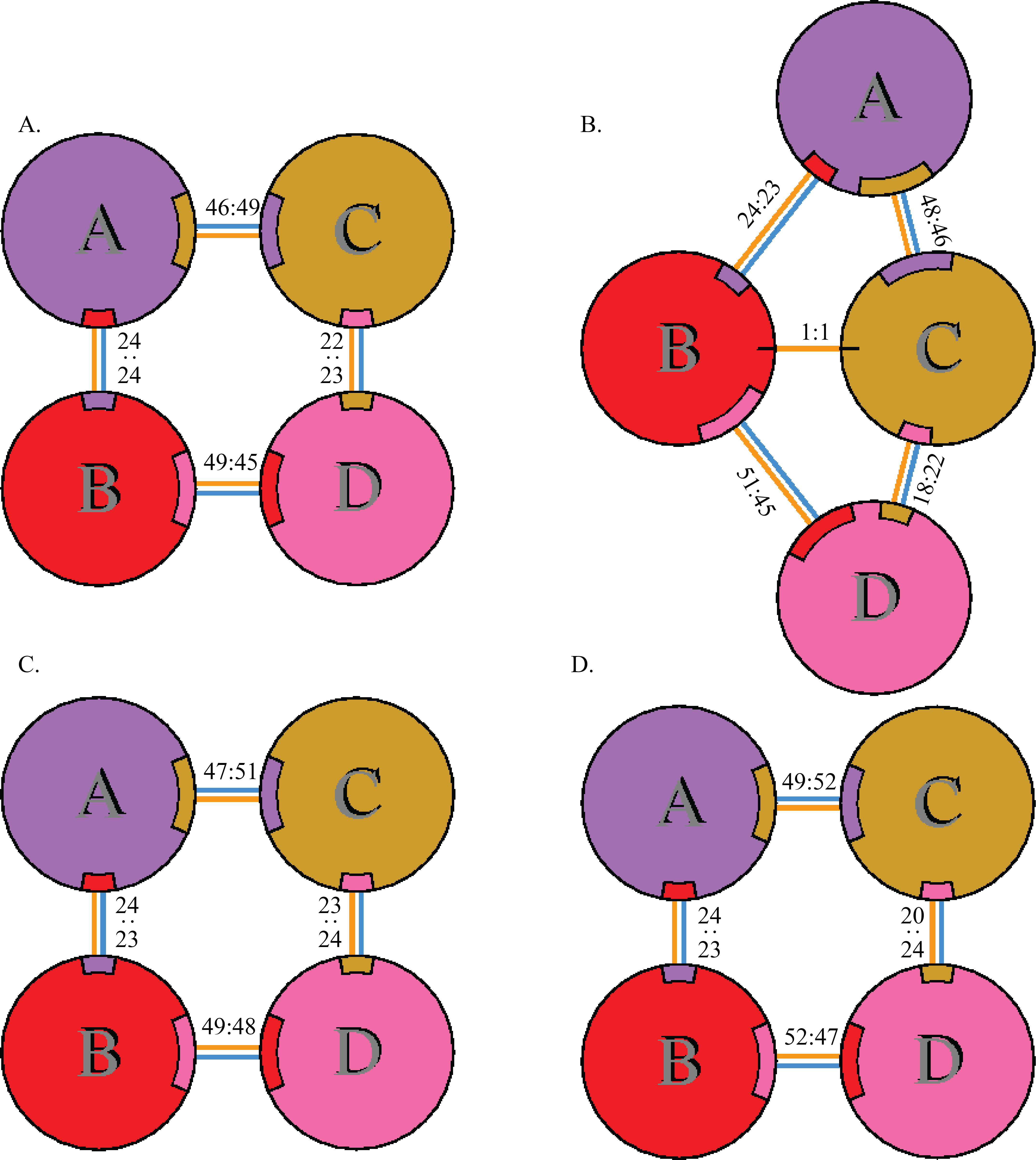

Supplement: S3 Fig — (A) Interface interaction in wild type PKM2 (B) Interface interaction in C31F nsSVP of PKM2 (C) Interface interaction in Q310P nsSVP of PKM2 (D) Interface interaction in S437Y nsSVP of PKM2. (TIF) [file pone.0120469.s003.tif]

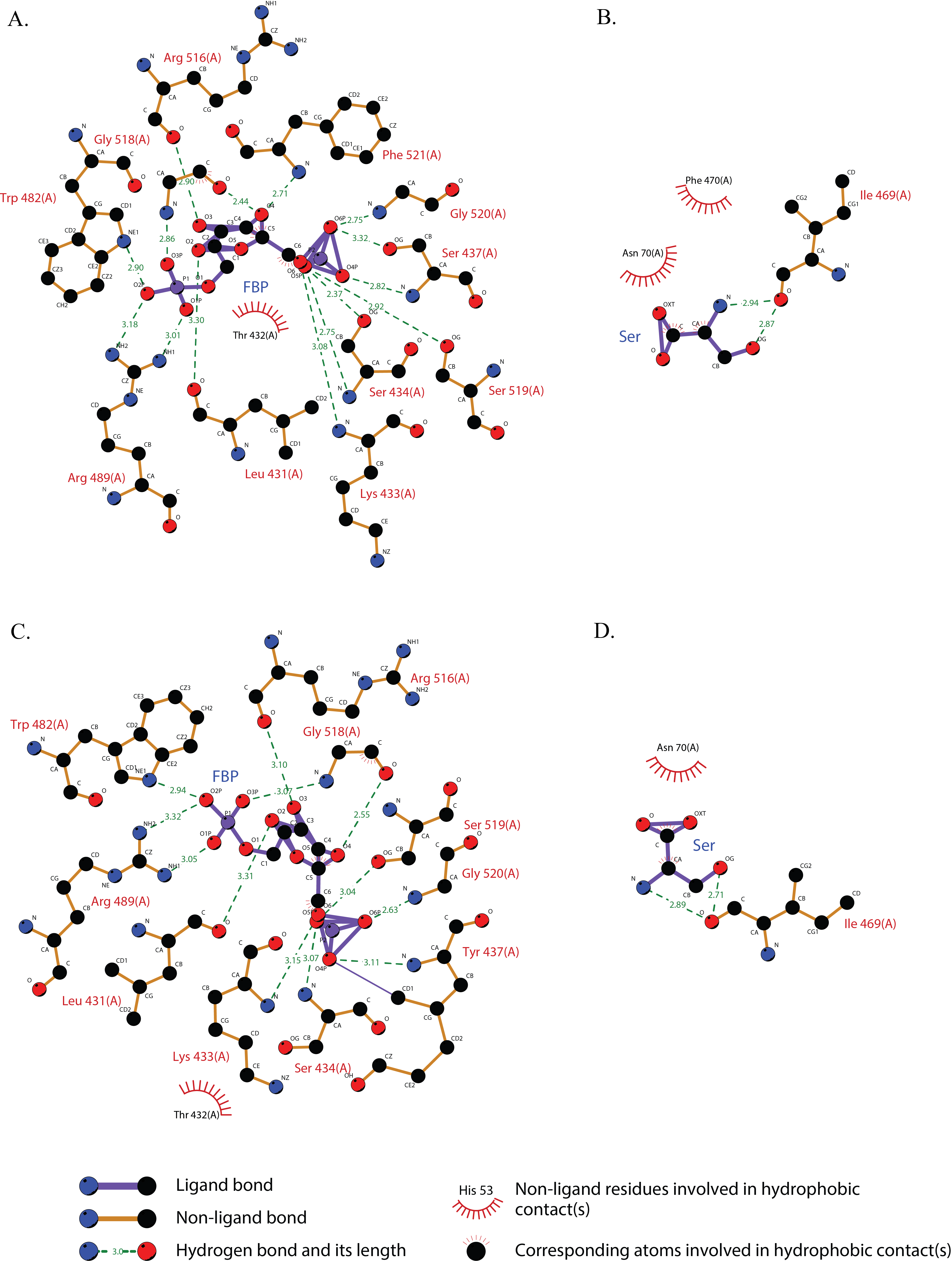

Supplement: S4 Fig — (A) Interaction between wild type PKM2 and FBP (B) Interaction between wild type PKM2 and L-Serine (C) Interaction between S437Y nsSVP and FBP (D) Interaction between S437Y nsSVP and L-Serine. (TIF) [file pone.0120469.s004.tif]
