# Supplementary material for: In Silico Screening, Genotyping, Molecular Dynamics Simulation and Activity Studies of SNPs in Pyruvate Kinase M2
Source: PLoS One. 2015 Mar 13;10(3):e0120469. doi: 10.1371/journal.pone.0120469 (PMC4359060; doi:10.1371/journal.pone.0120469)
Supplement: S1 Table — (DOCX) [file pone.0120469.s005.docx]

**Table S1.** Dimeric PKM2 stability analayis of functionally important nsSNPs

| **Energy** | **WILD** | **C31F** | **Q310P** | **S437Y** |
| --- | --- | --- | --- | --- |
| Total energy | -128.578 | -99.8632 | -78.9835 | -91.6853 |
| Backbone Hbond | -798.146 | -780.132 | -791.204 | -785.473 |
| Sidechain Hbond | -351.972 | -335.085 | -330.672 | -345.579 |
| Van der Waals | -1288.11 | -1271.74 | -1285.41 | -1284.51 |
| Electrostatics | -67.0678 | -71.055 | -64.9557 | -74.1094 |
| Solvation Polar | 1749.62 | 1723.64 | 1739.07 | 1748.67 |
| Solvation Hydrophobic | -1695.6 | -1677.15 | -1695.76 | -1691.46 |
| Van der Waals clashes | 26.5169 | 26.1069 | 35.9615 | 29.871 |
| entropy sidechain | 681.824 | 664.657 | 674.574 | 682.241 |
| entropy mainchain | 1632.61 | 1631 | 1654.99 | 1642.05 |
| torsional clash | 10.7182 | 12.4404 | 11.7054 | 13.1522 |
| backbone clash | 705.645 | 691.51 | 702.315 | 702.732 |
| helix dipole | -31.5282 | -25.5183 | -29.5035 | -29.2124 |
| energy Ionisation | 2.56168 | 2.96765 | 2.22764 | 2.67222 |
